# Finding Balance in Cortical Networks

**DOI:** 10.1371/journal.pbio.1001035

**Published:** 2011-03-22

**Authors:** Robin Meadows

**Affiliations:** Freelance Science Writer, Fairfield, California, United States of America

**Figure:**
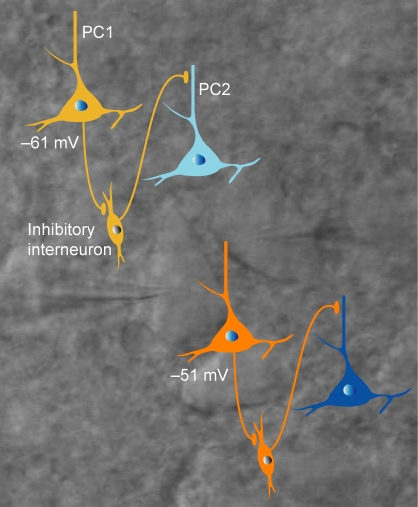
The amount of inhibition (light and dark blue) received by neocortical pyramidal cells is regulated by the membrane potential of nearby pyramidal cells. The background shows a paired recording from pyramidal cells.

No matter what you're doing at any given moment, from walking to talking or even sleeping, your brain is doing its own thing. Networks of neurons constantly and often spontaneously generate rhythmic electrical activity in the cortex, the brain's outermost layer and the seat of judgment, decision making, and other higher order functions. These cortical networks contain pyramidal cells that can excite inhibitory interneurons, which can in turn decrease the activity of pyramidal cells. This interplay of excitation and inhibition generates specific activity patterns that are critical to various cortical functions, like working memory and attention. However, it is not clear what cellular mechanisms maintain the proper balance between these two opposing inputs.

Now, in this issue of *PLoS Biology*, Yousheng Shu and colleagues report that small changes in the electrical properties of pyramidal cells help maintain the excitation–inhibition balance that keeps these cortical networks humming along. In addition to exciting other neurons via “all or none” events (also known as digital mode) called action potentials, pyramidal cells may also have another way of communicating within a network. The researchers had previously found that pyramidal cells can use a “graded” method (analog mode) of exciting their targets via small changes in their membrane potential. Because pyramidal cells activate inhibitory interneurons and thus generate recurrent inhibition, the researchers asked whether this analog control of membrane potential could fine-tune the balance between excitation and inhibition in the cortex.

The researchers began investigating recurrent network activity by recording activity between pairs of nearby pyramidal cells that presumably had an inhibitory interneuron between them. They first established that electrically stimulating one pyramidal cell in this microcircuit resulted in a late-onset, “slow” recurrent inhibition in the second pyramidal cell. They next made a positive (depolarizing) shift of the membrane potential by injecting current into the first pyramidal cell, and found that this increased the slow recurrent inhibition in the second cell. This modulation was sensitive to membrane potential shifts as small as 5 to 10 mV, considerably less than the shifts required to generate an all-or-none action potential.

What about the other connections in this microcircuit? Knowing that low-threshold spiking (LTS) interneurons can mediate slow recurrent inhibition, the authors next asked whether modulation of pyramidal cells can directly influence these inhibitory cells. Indeed, they found that small membrane potential shifts in pyramidal cells can modulate LTS interneuron activity. Importantly, they also observed these analog effects for fast spiking interneurons, which mediate “fast” recurrent inhibition. Taken together, these findings demonstrate that the membrane potential of pyramidal cells modulates recurrent inhibition, which helps balance the excitation and inhibition that lend stability to cortical network rhythms.

Finally, the researchers examined the possible mechanisms of this membrane potential effect, and found a role for a type of potassium current called the D-current that helps control the duration of axonal action potentials. Blocking the D-current with drugs increased the inhibitory effect between pairs of pyramidal cells as well as the excitatory effect of pyramidal cells on LTS interneurons. Based on these findings, the researchers proposed the following model: depolarization in the first pyramidal cell inactivates D-current and so prolongs axonal action potentials, thereby enhancing synaptic transmission to the interneuron that then causes more inhibition to the second pyramidal cell.

By showing that membrane potential helps balance excitation and inhibition in microcircuits, this work suggests a key role of analog communication in the rhythmic activity of cortical networks. Notably, recent work has implicated disruptions in the balance of excitation–inhibition in neurological disorders such as epilepsy and schizophrenia. Whether analog modulation may prove relevant to such diseases, however, is unknown. Many questions must be investigated before such possibilities can be addressed, including whether analog modulation applies to all cortical circuits, and whether it occurs during behaviorally relevant processes.

**Zhu J, Jiang M, Yang M, Hou H, Shu Y (2011) Membrane Potential-Dependent Modulation of Recurrent Inhibition in Rat Neocortex. doi:10.1371/journal.pbio.1001032**

